# Correlates of smoking quit attempts: Florida Tobacco Callback Survey, 2007

**DOI:** 10.1186/1617-9625-5-10

**Published:** 2009-06-29

**Authors:** Evelyn P Davila, Wei Zhao, Margaret Byrne, Monica Webb, Yougie Huang, Kristopher Arheart, Noella Dietz, Alberto Caban-Martinez, Dorothy Parker, David J Lee

**Affiliations:** 1 Department of Epidemiology and Public Health, University of Miami, USA; 2 Sylvester Biostatistics Core Resource, University of Miami, Miami, FL, USA; 3 Department of Psychology, University of Miami, Miami, FL, USA; 4 Florida Department of Health, Bureau of Epidemiology, Tallahassee, FL, USA; 5 Disparities and Community Outreach Core, University of Miami Sylvester Comprehensive Cancer Center, Miami, FL, USA

## Abstract

**Objective:**

The public health burden of tobacco-associated diseases in the USA remains high, in part because many people's attempts to quit are unsuccessful. This study examined factors associated with having lifetime or recent attempts to quit smoking among current smokers, based on a telephone survey of Florida adults.

**Methods:**

Data from the 2007 telephone-based Florida Behavioral Risk Factor Surveillance System (BRFSS) and its follow-up survey, the Tobacco Callback Survey, were used to assess determinants of having ever attempted to quit smoking and attempted to quit smoking in the past 12 months. All analyses were conducted using SAS.

**Results:**

Among 3,560 current smokers, 41.5% reported having tried to quit smoking in the past 12 months while 83.4% reported having ever tried to quit. Having a history of a tobacco-related medical condition was significantly associated with both recent (Adjusted Odds Ratio (AOR) 1.41 [Confidence Interval 1.19–1.65]) and lifetime quit attempts (AOR 1.43 [1.15–1.79]). Greater nicotine dependence and being advised by a physician to quit smoking were also positively associated with lifetime quit attempts.

Receipt of healthcare provider advice to quit smoking in the past 12 months and a strong belief that quitting following a long history of regular smoking would not result in health benefits and belief that there are health benefits to quitting smoking were associated with lifetime quit attempts.

**Conclusion:**

Targeted smoking cessation interventions are needed for smokers with selected medical conditions and with high nicotine dependence. The importance of physician advice in encouraging individuals to quit is further highlighted.

## Introduction

The 1964 Surgeon General's report on the effects of cigarette smoking on respiratory and cardiovascular health prompted interest in ways to decrease the prevalence of smoking [1], which has been a prominent public health goal since the report was issued. However, cigarette smoking continues to be a major determinant of poor health in the United States. Although the prevalence of smoking has declined since the 1964 report [1,2], 43 million adult smokers in the United States continue to smoke, which represents 20% of the adult population [3]. Research suggests that smoking and secondhand smoke exposure combined were responsible for 438,000 premature deaths, 5.5 million years of potential life lost, and over $90 million productivity losses in the United States each year for the period between 1997 and 2001 [4].

Over the years there has been a great deal of research on smoking cessation, including research on predictors of smoking cessation and successful quit attempts. However, given that intention to quit smoking is shown to be one of the key steps in the process towards smoking cessation [5], it is important to study all quit attempts, even those that are not successful [6]. Furthermore, the fact that these individuals are at least trying to quit smoking suggests that they are motivated, but are just unable to maintain long-term abstinence. In order to adequately address all barriers to smoking cessation among these smokers and increase the proportion of successful quit attempts, it is important to understand the characteristics of smokers who have unsuccessfully tried to quit smoking. There is also a sense of urgency with regard to how soon these smokers are reached, particularly since having many failed attempts may result in frustration, fear, defiance, and loss of interest in quitting [7].

Most studies that address determinants of quit attempts are based on specific populations such as adolescents or young adults [8-10], hospital/clinic and/or chronically-ill patients [9,11-14], specific race/ethnic background, or non-US populations [15-19]. Few studies have assessed quit attempts in the general population [20,21]. However, these studies, as well as the others in specific populations, have not undertaken comprehensive analysis of the many factors thought to be associated with quit attempts. The lack of such comprehensive analyses limits the identification of factors most strongly associated with quit attempts. Although studies have investigated the relationship between socio-demographic factors and quit attempts, these studies do not adjust for other factors that may play a role in quit attempts. For example, studies have shown that factors such as physician advice to quit smoking [16,17,22], smoking risk perception [14], nicotine dependence [13,14,20,23,24], weight concern [25,26], and history or presence of a tobacco-related condition [12,13,27] are associated with quit attempts. In addition, some of these studies on smoking quit attempts are based on individuals who have successfully quit smoking, and thus factors are assessed in relation to successful quit attempts. However, these factors may not relate in the same fashion with unsuccessful quit attempts. The purpose of this study was to thoroughly assess factors associated with having ever or recently (i.e., within the past 12 months) attempted to quit smoking among current smokers, using data from a sample of Florida residents in 2007.

## Methods

### Description of data set and sample

The Behavioral Risk Factor Surveillance System (BRFSS) is a nationwide state-based telephone survey developed to gather data on risk factors for morbidity and mortality among the non-institutionalized, civilian US population 18 years of age and older using disproportionate stratified random sampling. In 2007 the Florida Department of Health conducted a Florida Tobacco Callback Survey with smokers identified in the BRFSS survey who gave callback permission. The Tobacco Callback Survey included questions about smoking patterns, quit attempts, perceived benefits of quitting, and smoking frequency and nicotine dependence. Of the 8,230 current smokers identified in the BRFSS, 6,007 (73.0%) agreed to be re-contacted of which 2,310 (28.1%) either were not able to be contacted after 15 phone calls made or gave a firm refusal and 137 (1.7%) reported that they were no longer smoking and were therefore ineligible to participate. Thus, the analyses for this paper are based on the 3,560 (43.3%) of the BRFSS smokers who agreed to be contacted, were successfully called and re-contacted, were still smoking, and completed the follow-up interview with no missing values of age and race.

In this study, data from the 2007 Florida BRFSS and Florida Tobacco Callback Survey were one to one match-merged by participant sequential number. Using data from the BRFSS, we compared the socio-demographic and selected health characteristics of the smokers who did and did not participate in the Tobacco Callback Survey and found differences that were of small magnitude (Table 1). In assessing whether any differences were meaningful, we calculated the effect sizes for selected variables between the respondents and non-respondents. Effect sizes were all <0.2, which is considered to be small effect[28]

**Table 1:** Demographics and SES of current smoker BRFSS vs. Callback survey

	BRFSS		Callback		
	
Demographics and SES	n	%	n	%	Effect^a^
Total participants	4670	100.0	3560	100.0	
**Age in years**					
Missing	39	0.8	12	0.3	1.03
18 – 39 yrs	1122	24.0	690	19.4	0.11
40 – 54 yrs	1712	36.7	1335	37.5	0.02
55 or older	1797	38.5	1523	42.8	0.09
**Gender**					
Male	1973	42.2	1314	36.9	0.11
Female	2697	57.8	2246	63.1	0.11
**Race/Ethnicity**					
Missing	34	0.7	18	0.5	0.46
Non-Hispanic White	3729	79.9	2980	83.7	0.10
Non-White or Hispanic	907	19.4	562	15.8	0.09
**Education level**					
Missing	30	0.6	5	0.1	1.01
Did not graduate High School	796	17.0	595	16.7	0.01
Graduated High School	1827	39.1	1304	36.6	0.06
Attended College or Technical School	1270	27.2	1100	30.9	0.08
Graduated from College or Technical School	747	16.0	556	15.6	0.01
**Income**					
Missing	639	13.7	346	9.7	1.67
Less than $15,000	627	13.4	571	16.0	0.06
$15,000 to less than $25,000	998	21.4	808	22.7	0.03
$25,000 to less than $35,000	617	13.2	478	13.4	0.01
$35,000 to less than $50,000	664	14.2	516	14.5	0.01
$50,000 or more	1125	24.1	841	23.6	0.01
**Have any health care coverage**					
Not asked or Missing	10	0.2	4	0.1	0.27
No	1262	27.0	917	25.8	0.03
Yes	3398	72.8	2639	74.1	0.03
**Marital status**					
Missing	23	0.5	5	0.1	0.74
Married	2026	43.4	1608	45.2	0.04
Divorced, widowed, separated	1812	38.8	1460	41.0	0.05
Others (never married, unmarried couple)	809	17.3	487	13.7	0.10
**Children in household**					
Missing	23	0.5	5	0.1	0.74
No	3151	67.5	2427	68.2	0.01
Yes	1496	32.0	1128	31.7	0.01
**Body Mass Index**					
Missing	226	4.8	59	1.7	0.67
Neither overweight nor obese	2017	43.2	1458	41.0	0.05
Obese	931	19.9	858	24.1	0.03
Overweight	1496	32.0	1185	33.3	0.10
**Weight change in the past year**					
Missing	301	6.4	101	2.8	0.72
Gain/loss < = 3 kg	2668	57.1	1950	54.8	0.05
Loss > 3 kg	936	20.0	816	22.9	0.07
Gain >3 kg	765	16.4	693	19.5	0.08
**Intentional weight change**					
Missing	28	0.6	23	0.6	0.10
No change	2396	51.3	1588	44.6	0.13
Intentional	783	16.8	687	19.3	0.07
Not intentional	1463	31.3	1262	35.4	0.09
**Ever had any medical condition**					
No	2573	55.1	1757	49.4	0.12
Yes	2097	44.9	1803	50.6	0.12
**Binge drinking**					
Missing	343	7.3	71	2.0	1.07
No	3374	72.2	2817	79.1	0.16
Yes	953	20.4	672	18.9	0.04
**Heavy alcohol consumption**					
Missing	347	7.4	82	2.3	1.03
No	3778	80.9	3072	86.3	0.14
Yes	545	11.7	406	11.4	0.01

^a ^Effect sizes between two independent proportion (BRFSS and Callback).

### Definitions and statistical analyses

Variables for recent and lifetime attempt to quit smoking were defined based on two questions in the Tobacco Callback Survey. Having a recent quit attempt was indicated when a respondent answered "yes" to the question of "Have you tried to quit smoking completely during the past 12 months." A lifetime quit attempt was defined when a respondent answered "yes" to the question of "Have you ever tried to quit smoking completely." Factors that are potentially associated with attempts to quit were selected based on literature review on successful quit attempts. These variables included demographic characteristics (e.g., age, race/ethnicity, gender, marital status, educational attainment, number of children), average of number of days per month smoked, number of cigarettes smoked per day, perceived health benefit of quitting based on the question "If a person has smoked a pack of cigarettes a day for more than 20 years, there is little health benefit to quitting smoking," body mass index (BMI), weight change, healthcare provider advice to quit smoking, nicotine dependence, and history of a tobacco-related medical condition.

Nicotine dependence was a composite variable derived from a factor analysis (6 items, score range 1–4, *α *= 0.81, a higher score indicates more nicotine dependence, then classified into 3 levels: heavier dependence if score > = 5, moderate dependence if 2< score <5 and lighter dependence if score < = 2) which included items such as: "Do you have trouble going more than a few hours without smoking;" "Even in a bad rainstorm, if you ran out of cigarettes, you would probably go to the store to get some more"; "When you go without smoking for a few hours, you experience craving." The questions used for our definition of nicotine dependence are based on slight modifications of questions from the Nicotine Dependence Syndrome Scale (NDSS) [29]. The measure for a history of tobacco-related medical condition (Yes/No) was created based on self report of any of the following medical conditions: diabetes, high blood pressure, heart attack, coronary heart disease (CHD), angina, stroke, and asthma. If the participant answered yes to any of these medical conditions they were considered to have a "tobacco-related medical condition." All variables used in the present study are based on self-reported data as BRFSS is a survey administered via the telephone.

Descriptive statistics of categorical data are presented as percentages, and continuous data are presented as means and standard deviations (SD). Simple logistic regressions were first conducted for all the potential independent variables. To reduce the possibility of confounding in the multiple regression models, variables with a p-value less than or equal to 0.20 in simple logistic regression models were included in initial multiple logistic regression models simultaneously to identify factors independently associated with recent and lifetime smoking quit attempts [30]. Age, gender and race/ethnicity were included in the models regardless of the significance level. Covariates were assessed for pre-specified interactions. Comparisons resulting in a p-value of 0.05 or less were considered to be statistically significant. SAS version 9.2 (SAS Institute, Inc; Cary, NC) was used for all of the analyses. This study was approved by the University of Miami Institutional Review Board.

## Results

The sample characteristics are shown in Table 2. The majority of the 3,560 current smokers in the Tobacco Callback Survey reported their race as White (84.1%) with a slightly greater proportion of females than males (63.1%). Over 83% of the participants reported a high school education or above. More than 58% were overweight or obese using standard definitions. Approximately 42% reported to have tried to quit smoking completely during the past 12 months while about 83% reported to have ever tried to quit smoking completely. More than 50% of these smokers reported ever having a tobacco-related medical condition.

**Table 2:** Sample Characteristics: Florida Tobacco Callback Survey, Behavioral Risk Factor Surveillance System, 2007 (n = 3560)

Variable	n	%
**Age in years**		
**18 – 39 yrs**	690	19.5
40 – 54 yrs	1335	37.6
> = 55 yrs	1523	42.9
**Gender**		
Female	2246	63.1
**Race/Ethnicity**		
White, non-Hispanic	2980	84.1
Black, non-Hispanic	217	6.1
Hispanic	156	4.4
Other race, non-Hispanic	189	5.3
**Education level**		
Did not graduate High School	595	16.7
Graduated High School	1304	36.7
Attended College or Technical School	1100	30.9
Graduated from College or Technical School	556	15.6
**Marital status**		
Married	1608	45.2
Divorced, widowed, separated	1460	41.1
Others (never married, unmarried couple)	487	13.7
**Children in household**	1128	31.7
**Body Mass Index**		
Neither overweight nor obese	1458	41.7
Overweight	1185	33.9
Obese	858	24.5
**Weight change in the past year**		
Gain/loss < = 3 kg	1950	56.4
Loss > 3 kg	816	23.6
Gain >3 kg	693	20.0
**Intentional weight change**		
No change	1588	44.9
Intentional	687	19.4
Not intentional	1262	35.7
**Ever had any medical condition**	1803	50.7
**Binge drinking**	672	19.3
**Heavy alcohol consumption**	406	11.7
**Nicotine dependence**		
Lighter dependence	1295	38.8
Moderate dependence	910	27.3
Heavier dependence	1134	34.0
**Heath care provider advice to stop smoking in the past 12 months**		
No/Non visit	1285	38.2
Yes	2079	61.8
**Perceived benefit of quitting: " If long time smoker, little health benefit to quitting"**		
Strongly agree	241	7.4
Agree	625	19.2
Disagree	1368	42.1
Strongly disagree	1018	31.3
**Tried to quit smoking completely during the past 12 months**	1409	41.5
**Ever tried to quit smoking completely**	2808	82.7
	**Mean**	**std**
**Number of years smoked**	33.6	14.3
**Number of cigarettes smoked per day**	18.6	11.0
**Number of days smoked cigarettes in the past 30 days**	27.6	6.6
**Number of cigarettes smoked per day in the past 30 days**	17.4	11.1

There were no significant first order interactions between any of the variables, including the variables "ever had a tobacco-related medical condition" and "provider advice for quitting smoking." Thus, no interaction terms were included in the final regression models.

### Correlates of quit attempts in the past 12 months

Females were slightly but significantly more likely to report a quit attempt in the past 12 months than males (Adjusted Odds Ratio [AOR] 1.19, [95% Confidence Interval, 1.00–1.40]); relative to non-Hispanic Whites, non-Hispanic Blacks were also more likely to make a quit attempt (AOR 1.63 [1.15–2.30]) (Table 3). Compared to adults 18–39 years of age, adults 40–54 years of age and 55 years of age and older were less likely to report a quit attempt in the previous 12 months (AOR 0.79 [0.63–0.99]; and AOR 0.71 [0.54–0.93], respectively). Smokers who graduated from high school were less likely to have reported a recent quit attempt compared to smokers who did not graduate from high school (AOR 0.78 [0.62–0.99]). Compared to those reporting no weight changes, smokers who reported a weight loss of 3 kg or greater in the previous 12 months were more likely to have made a quit attempt during the previous 12 months (AOR 1.38 [1.04–1.82]). Report of at least one tobacco-related medical condition was associated with increased odds of a recent quit attempt (AOR 1.41 [1.19–1.65]). Smokers who received healthcare-provider advice to quit smoking in the past 12 months were more likely to report a quit attempt during the same time period (AOR 1.53 [1.30–1.81]). Both the number of days smoking and the amount smoked in the previous 30 days were associated with lower odds of a 12-month quit attempt.

**Table 3:** Multiple Logistic Regression of Smoking Quit Attempts, Florida Tobacco Callback Survey, Behavioral Risk Factor Surveillance System, 2007^a^

	Smoking Quit Attempt							
	
	Past 12 months n = 2917 Yes = 1230 (42.2%)				Lifetime n = 2915 Yes = 2429 (83.3%)			
**Variable**	**AOR^b^**	**L95^c^**	**U95^d^**	**P-value**	**AOR**	**L95**	**U95**	**P-value**

**Age in years**								
18 – 39 yrs	1.000				1.000			
40 – 54 yrs	0.787	0.625	0.991	0.0417	1.572	1.185	2.084	0.0017
> = 55 yrs	0.706	0.538	0.926	0.0119	1.290	0.960	1.733	0.0914
**Sex**								
Male	1.000				1.000			
Female	1.186	1.002	1.402	0.0470	1.029	0.828	1.278	0.7986
**Race/ethnicity**								
White-Non-Hispanic	1.000				1.000			
Black-Non-Hispanic	1.625	1.148	2.299	0.0061	0.793	0.513	1.226	0.2969
Hispanic	1.025	0.684	1.537	0.9039	0.607	0.384	0.960	0.0328
Other race, Non-Hispanic	1.099	0.785	1.538	0.5843	1.047	0.663	1.653	0.8430
**Education level**								
Did not graduate High School	1.000				1.000			
Graduated High School	0.779	0.615	0.986	0.0382	1.013	0.755	1.359	0.9320
Attended College or Technical School	0.840	0.658	1.073	0.1621	1.439	1.044	1.984	0.0264
Graduated from College or Technical School	0.767	0.577	1.021	0.0688	1.128	0.782	1.627	0.5197
**Marital status**								
Married	1.000				1.000			
Divorced, widowed, separated	1.140	0.962	1.351	0.1299	0.800	0.638	1.004	0.0538
Others (never married, unmarried couple)	1.007	0.782	1.297	0.9567	0.773	0.568	1.051	0.1003
**Children in household**								
Yes vs. No	1.039	0.855	1.264	0.6981	NA^e^			
**Weight change the past year**								
Gain/loss < = 3 kg	1.000				1.000			
Loss > 3 kg	1.377	1.043	1.817	0.0242	1.206	0.831	1.749	0.3243
Gain >3 kg	1.288	0.978	1.696	0.0716	1.024	0.716	1.464	0.8978
**Intentional weight change**								
No change	1.000				1.000			
Intentional	1.088	0.802	1.475	0.5894	1.070	0.715	1.599	0.7426
Not intentional	1.163	0.907	1.492	0.2339	0.954	0.694	1.313	0.7730
**Ever had tobacco-related medical condition**								
No	1.000				1.000			
Yes	1.405	1.193	1.654	<.0001	1.433	1.154	1.779	0.0011
**Binge drinking**								
No	1.000				NA			
Yes	0.883	0.702	1.111	0.2888				
**Heavy alcohol consumption**								
No	1.000				1.000			
Yes	1.023	0.773	1.353	0.8746	0.821	0.606	1.112	0.2032
**Nicotine dependence**								
Lighter dependence	NA				1.000			
Moderate dependence					1.526	1.174	1.984	0.0016
Heavier dependence					1.827	1.387	2.404	<.0001
**Heath care provider advice to stop smoking in the past 12 months**								
No/no visit	1.000				1.000			
Yes	1.534	1.302	1.807	<.0001	1.564	1.273	1.922	<.0001
**Perceived benefit of quitting: "If long time smoker, little health benefit to quitting"**								
Strongly agree	1.000				1.000			
Agree	0.785	0.557	1.107	0.1680	0.799	0.537	1.189	0.2689
Disagree	0.909	0.661	1.251	0.5585	1.157	0.791	1.692	0.4528
Strongly disagree	1.217	0.878	1.687	0.2392	2.146	1.419	3.247	0.0003
**Number of cigarettes smoked per day**								
Every one cigarette increased	1.005	0.995	1.014	0.3195	1.007	0.994	1.021	0.2964
**Number of days smoked cigarettes in the past 30 days**								
Every one day increased	0.958	0.946	0.971	<.0001	NA			
**Number of cigarettes smoked per day in the past 30 days**								
Every one cigarette increased	0.988	0.978	0.998	0.0162	0.977	0.964	0.990	0.0006

### Correlates of Lifetime Quit Attempts

Odds of a lifetime history of at least one quit attempt was significantly higher in adults 40–54 years of age relative to adults 18–39 years of age (AOR 1.57 [1.19–2.08]) (Table 3). Relative to White non-Hispanics, Hispanic smokers were less likely to report a lifetime quit attempt (AOR 0.61 [0.38–0.96]) Smokers who had attended some college or technical school had a greater odds of a lifetime quit attempt relative to smokers who did not complete high school (AOR 1.44 [1.04–1.98]). Reports of at least one tobacco-related medical condition were associated with increased odds of an ever quit attempt (AOR 1.43 [1.15–1.78]). Compared to participants with lower levels of nicotine dependence, smokers with moderate and heavy dependence were more likely to have reported at least one quit attempt in their lifetime (AOR 1.53 [1.17–1.98]; and AOR 1.83 [1.39–2.40], respectively). Receipt of healthcare provider advice to quit smoking in the past 12 months was associated with increased odds of a lifetime quit attempt (AOR 1.56 [1.27–1.92]). Compared to those who strongly believed that quitting following a long history of regular smoking would not result in health benefits, smokers who strongly disagreed with this statement were more likely to report a lifetime quit attempt (AOR 2.15 [1.42–3.25]. Finally, the odds of a lifetime quit attempt were inversely associated with the number of cigarettes smoked in the past 30 days.

## Discussion

Most smokers have thought about quitting smoking at one point or another, for various reasons. We found that about eight in 10 Florida smokers have attempted to quit at some point in their lifetime, while four in 10 Florida smokers reported having recently tried to quit. However, the fact that they are still smoking highlights the difficulty most smokers have with achieving successful cessation as well as the importance of characterizing individuals who at least attempt to quit, since they may be more likely to benefit from smoking cessation programs. In this study, we assessed factors associated with ever attempting to quit smoking and recent quit attempts among a sample of current adult smokers in Florida.

In the present study, older smokers were more likely than younger smokers to have ever attempted quitting smoking but were less likely to have tried quitting in the past 12 months, even after controlling for nicotine dependence, perceived benefits of quitting, smoking risk perception, and history or presence of tobacco-related medical conditions. This finding is consistent with another US population based survey of smokers which found older smokers to be least likely to attempt quitting in the past year and be less likely to be successful at quitting [20]. A potential explanation for the difference in quit attempts by age group could be due to resistance to smoking cessation advice due to difficulty quitting, particularly those with many failed attempts, as well as due to changes in social norms regarding tobacco use [20]. It could also be that young smokers try to quit smoking more frequently than older smokers; however the number of quit attempts is not known. We also found that non-Hispanic blacks were the most likely to have reported a quit attempt in the past 12 months while Hispanics were the least likely to have tried quitting smoking in their lifetime, even after controlling for potential confounders. A US population based survey also found blacks to be more likely to report a recent quit attempt although they were least likely to be successful [20]. Again, racial/ethnic differences in quit attempts could be related to social and cultural norms as well as frequency of quit attempts.

The results also showed that history of having a tobacco-related medical condition was associated with greater likelihood of having attempted to quit smoking, both in the last 12 months or in their lifetime. This finding is not entirely surprising given that one reason individuals give for attempting to quit is their health [17,31,32]. Furthermore, when smokers have been asked if being diagnosed with a medical condition (e.g., heart disease) would increase their desire to quit smoking, the majority have agreed [12]. Studies have also shown a link between diagnosis of cardiovascular disease and increased motivation to quit smoking and smoking cessation [11,33,34]. In addition, nationally representative data do show the prevalence of smoking to be lower among adults with a history of stroke or myocardial infarction (18%) compared to adults without a history of cardiovascular events (26%) [35]. However, in a study of emergency department patients it was found that patients with a diagnosis of a cigarette-related medical condition (i.e., cardiovascular and respiratory diseases, peptic ulcer disease, and cancers such as of the lip, esophagus, lung, etc.) were less motivated to quit smoking when compared to smokers without such conditions [13]. Nevertheless, the desire to quit smoking may be contingent on whether the smoker believes their medical condition is related to their smoking, and/or whether they believe quitting will help their condition (since it is already present). In fact, in the present study we did find that smokers who perceive that there are health benefits to quitting smoking are more likely to have ever attempted to quit smoking, which is consistent with a study of emergency patients [14].

We also found that greater level of nicotine dependence was associated with being more likely to have ever attempted to quit smoking, although a statistically significant association was not observed for recent quit attempts. Although our findings are plausible given that greater nicotine dependence has been associated with readiness to quit smoking [14], our findings are inconsistent with other studies that have noted an inverse relationship between degree of nicotine dependence and quit attempts [20,24]. However, the differing findings could be related to difference in the definition of nicotine dependence. In agreement with previous studies however [24], we found greater number of smoked cigarettes per day in the past 30 days was inversely related to both recent and lifetime quit attempts. Although the association between nicotine dependence and quit attempts are not entirely clear, our findings suggest that highly dependent smokers are not giving up on quitting smoking and recognize the importance of quitting.

One of the very encouraging findings from our study was related to healthcare provider advice. In agreement with previous studies [16,17,22,36,37], we found that smokers who were advised by their healthcare provider to stop smoking were about 50% more likely to attempt to quit smoking, both in their lifetime and recently. Thus, consistent with the Public Health Service Clinical Practice Guidelines *Treating Tobacco Use and Dependence *[38], healthcare providers should continue to advise their patients to stop smoking, regardless of the frequency of smoking by their patients or other patient characteristics [36]. The importance of such advice is greatest for individuals with medical conditions, given the greater contact these smokers have with the healthcare system. In fact, increasing to 85% the proportion of healthcare professionals who counsel their high-risk patients (e.g., with coronary heart disease) about smoking cessation is one Healthy People 2010 goal [39].

Finally we found that those who lost more than 3 kg in the past year were more likely to have recently tried to quit smoking. This finding could be due to those with a medical condition (i.e. being medically ill), who lost weight because of their illness, deciding to quit smoking as part of their treatment or recovery regimen. Alternatively, it might be that these individuals are making an overall effort to improve their health, including both weight loss and smoking cessation attempts.

There are several limitations to this study. First, a number of variables that may also be related to quit attempts were not available in the dataset. These include extent of knowledge of the health effects associated with smoking, diagnosis of tobacco related cancers, diagnosis of mental health conditions such as depression, and the smoking status of the family and friends particularly if these individuals live with the participant [8,20,40]. Second, we do not know the extent of participants' awareness and perceived effectiveness of smoking cessation support, factors which have been associated with greater likelihood of a quit attempt [41]. It would have also been beneficial to know the frequency of quit attempts as well as the timing of these quit attempts. Finally, the results may not be generalized to other populations outside Florida or the United States.

## Conclusion

In conclusion, we found smokers who had a tobacco-related medical condition, who had greater nicotine dependence, who were advised by their physician to quit smoking, and/or who strongly believed that there are health benefits to quitting smoking were more likely to have smoking quit attempts, either in the past 12 months or ever. In addition, many older smokers appear have given up on quitting smoking given that they are more likely to have ever tried quitting smoking yet not in the past 12 months. However, Blacks and Hispanics appear have tried quitting smoking more recently than in the past. The present study further highlights the importance of awareness of the health implications associated with smoking, particularly if education is provided by a health care provider. Given blacks and older smokers may be less successful at quit smoking [20], these groups should be targeted for smoking cessation education. Further research is needed to understand the relationship between nicotine dependence, race/ethnicity, and quit attempts however. In addition, research is needed to characterize smokers with tobacco-related medical conditions and/or greater nicotine dependence in order to develop targeted smoking cessation interventions. Nevertheless, the information provided by this study can guide the development of targeted intervention programs for smokers who appear to want to quit.

## Competing interests

The authors declare that they have no competing interests.

## Authors' contributions

ED was the lead author of the manuscript. WZ managed the data and performed all statistical analyses. MB, MW, YH, KA, ND, AC, and DL assisted in the writing of the manuscript. YH and KA also provided statistical support. In addition, YH provided the data and assisted with data management issues. DL was the PI for the grant funding the study. All authors read and approved the final manuscript version.
